# Massive hematochezia due to jejunal varices successfully treated with coil embolization

**DOI:** 10.1002/ccr3.6339

**Published:** 2022-09-20

**Authors:** Sahar Nasr, Wafa Dahmani, Hanene Jaziri, Yesser Becheikh, Wafa Ben Ameur, Nour Elleuch, Ali Jmaa

**Affiliations:** ^1^ Department of Gastroenterology University of Sousse Sousse Tunisia; ^2^ Department of Radiology University of Sousse Sousse Tunisia

**Keywords:** cirrhosis, coil embolization, gastrointestinal bleeding, portal hypertension

## Abstract

We report a case of hematochezia with hemodynamic instability due to jejunal varices in a cirrhotic patient with no prior history of surgery. The patient was managed with coil embolization via the portal vein. After which, the patient did not present any hemorrhage recurrence and maintained a stable hemoglobin level.

## Graphical abstract

Coil embolization is a simple and safe procedure in patients presenting with ectopic varices‐related hemorrhage, especially when endoscopic treatment is unavailable or technically unfeasible. Yet, seen the high reported prevalence of rebleeding, decompressive treatments should always be discussed in a *multidisciplinary* team after coil embolization.

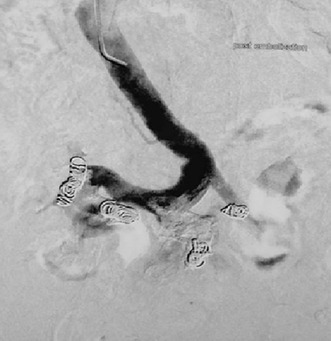

## 1 INTRODUCTION

Portal hypertension is a frequent complication of cirrhosis and it can cause a myriad of pathologic changes along the entire gastrointestinal (GI) tract including esophageal varices, gastropathy, and enteropathy. Among portal enteropathy manifestations, small bowel varices are the hardest to diagnose and the most life threatening.^1^ These varices develop to offer portal blood another way to reach the systemic circulation. They are defined by dilated portosystemic collateral veins developing anywhere in the abdomen but the cardio‐esophageal region. Small bowel varices are documented in up to 38.9% of patients with portal enteropathy and 5.3%–18.4% of cirrhotic patients.^1^, ^2^ Classically, small bowel varices occur in patients with prior history of abdominal surgery or at ostomy anastomosis.^3^

We report a case of a recurrent jejunal variceal bleeding in a patient without a prior history of abdominal surgery treated by percutaneous coil embolization and we review the current literature pertaining this condition.

## 2 CASE REPORT/CASE PRESENTATION

A 47‐year‐old woman with a history of cryptogenic Child‐Pugh C cirrhosis presented with a 3‐day hematochezia and melena. The patient had had five prior episodes of esophageal varices‐related GI bleeding treated with an association of non‐selective beta blockers and endoscopic band ligation.

Upon admission, her vital signs were as follows: a blood pressure of 80/50 mmHg, a heart rate of 110 bpm/min, and a respiratory rate of 22 cycle/min. Her abdomen was distended with shifting dullness and the spleen was palpable. The laboratory work‐up revealed decreased hemoglobin level (4.8 g/dl), decreased platelet count (81,000/mm^3^), decreased albumin level (25.5 g/dl), and prothrombin time level (25%). Initial diagnosis was a recurrence of esophageal varices bleeding. The patient immediately received intravenous (IV) esomeprazole, IV octreotide, and IV ceftriaxone and was transfused with packed red blood cells.

**FIGURE 1:**
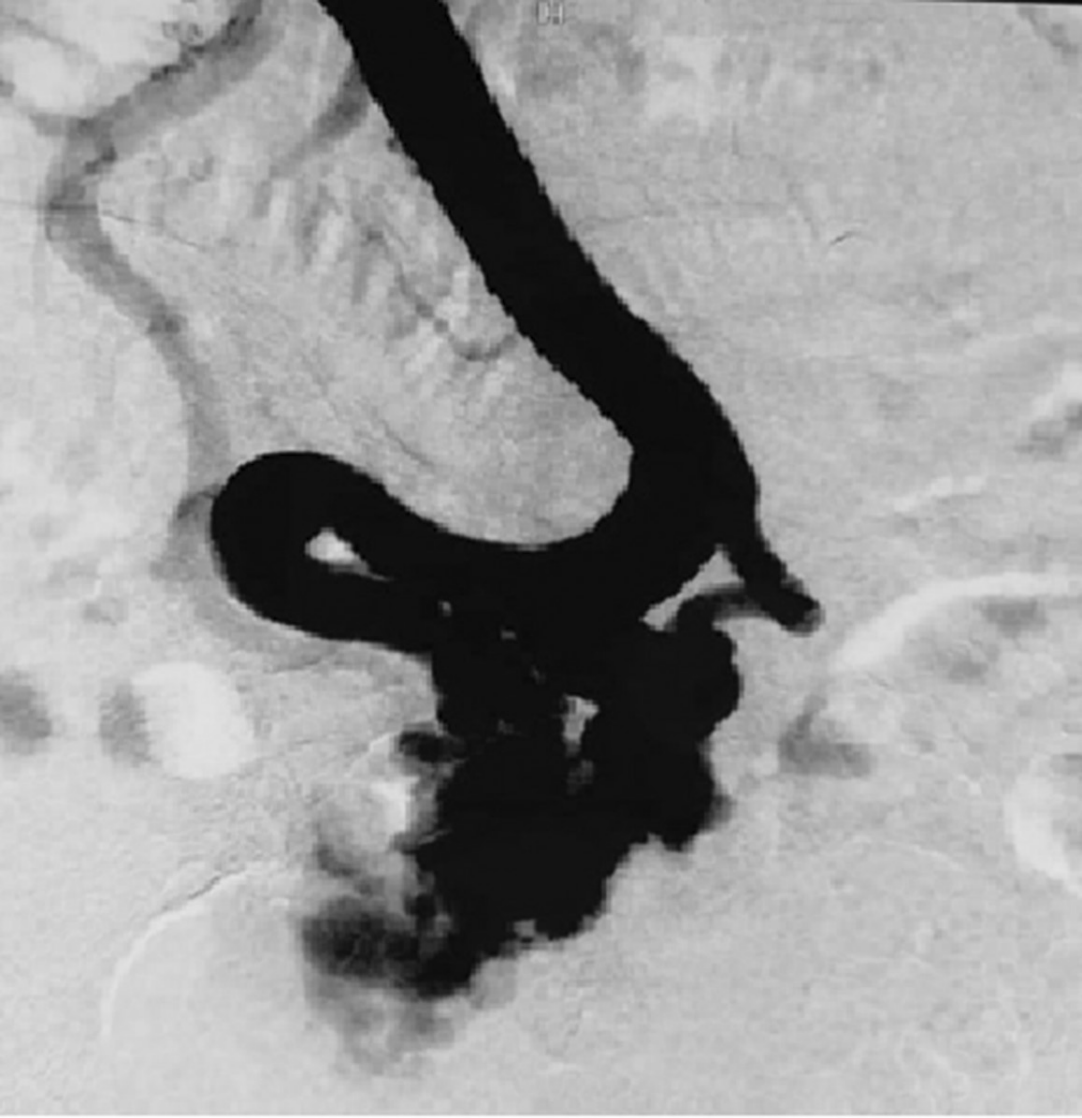
Portal venography showing jejunal varices

**FIGURE 2:**
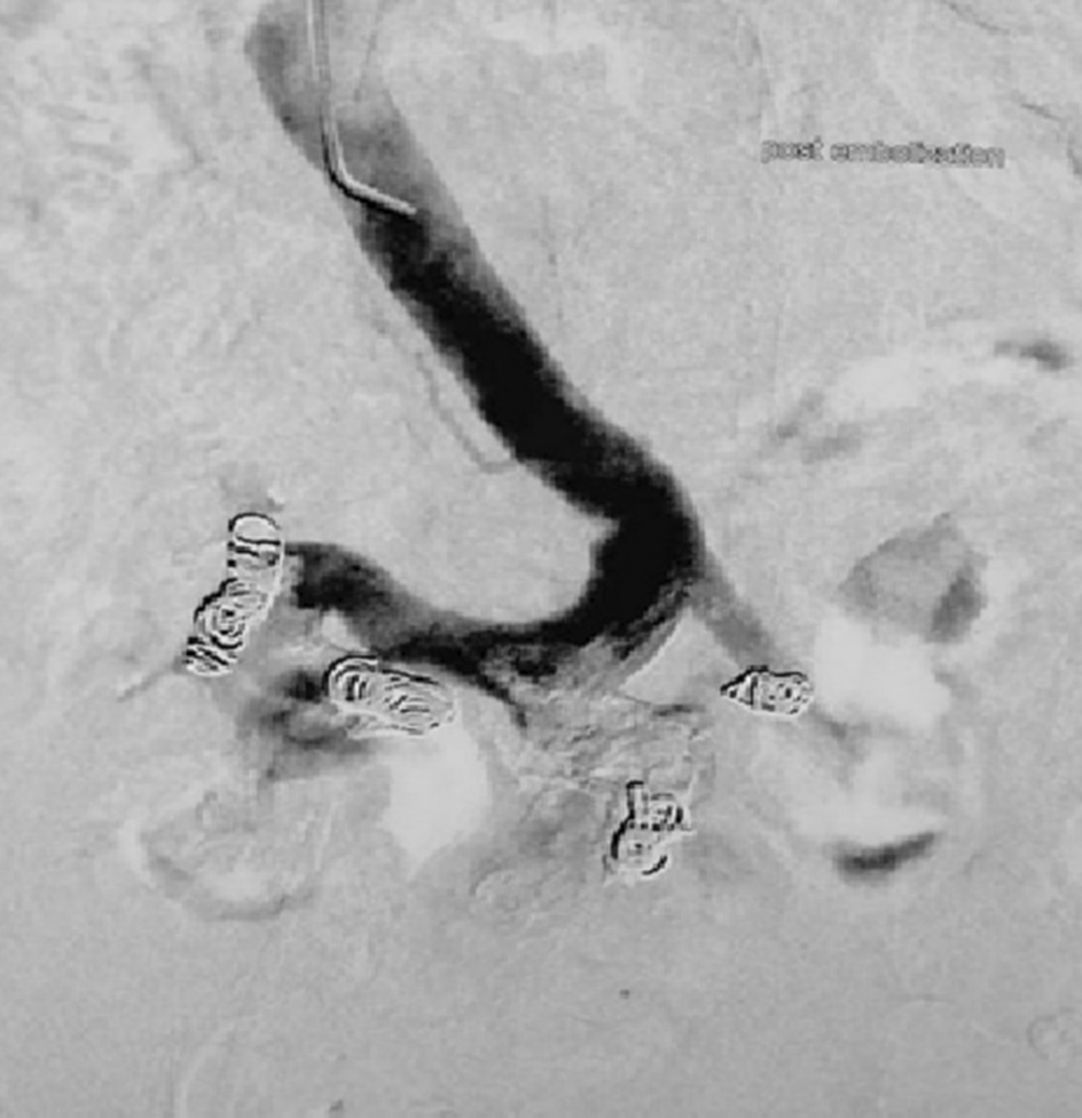
Venography post coil embolization of jejunal varices

Emergency esophagogastroduodenoscopy revealed grade I esophageal varices and stigma of bleeding in the duodenum. Abdominal CT demonstrated features of liver cirrhosis, mild ascites, a thrombosed portal vein involving extra‐ and intrahepatic segments and varices of the jejunal branch of the superior mesenteric vein that adhered to the abdominal wall. The interventional radiology team decided that transjugular intrahepatic portosystemic shunt (TIPS) would be difficult owing to portal vein partial obstruction in addition to the patient's advanced liver disease. Thus, the patient was initially managed conservatively. Seven days later, the patient presented a recurrence of hematochezia with hemodynamic instability requiring the use of vasopressor drugs. We decided to perform percutaneous varices embolization via the portal vein. Under local anesthesia, percutaneous transhepatic puncture of the intrahepatic branch of the portal vein was performed and a 5F catheter was inserted into the portal vein. Subsequently, the guidewire was manipulated into the superior mesenteric vein and the catheter tip was then positioned into tortuous, wide jejunal varices communicating with the right ovarian vein and drained to the inferior vena cava (Figure 1). Twenty steel coils were placed into these veins. The angiographic control following embolization showed a significant slowing down of blood flow (Figure 2). Following the procedure, the patient underwent regular clinical and biological follow‐up with iterative upper GI endoscopy to ensure esophageal varices eradication.

The patient was transfused with up to 14 units of packed red blood cells before the coiling embolization of the jejunal varices. Two years later, she has not presented any other bleeding episodes and has maintained a stable hemoglobin level.

## 3 DISCUSSION/CONCLUSION

Small intestine varices are a rare cause of GI hemorrhage. They are thought to account for 5% of all cases of varices‐related bleeding.^4^ Usually associated with PHT, small bowel varices represent potential passageways to relieve the increased portal venous flow. In fact, a Japanese study identified the presence of portosystemic shunts, in particular left gastric vein and splenorenal shunts as the only independent predictor of portal enteropathy.^5^ Furthermore, small bowel varices typically occur in patients with prior history of abdominal surgery or at ostomy anastomosis.^6^ In such cases, intestine walls where collateral circulation develops are usually adherent to old suture lines. Much more rarely, small bowel varices can be idiopathic. In fact, familial idiopathic small intestine varices have been reported previously.^7^ To the best of our knowledge, only three cases have been described.^7^, ^8^, ^9^ It is worth noting however that the case reports of idiopathic colic varices seem to be more common.^9^

Small bowel varices are reported to bleed in 25% of cases.^10^ The clinical presentation of small bowel varices is typically characterized by a triad: history of abdominal surgery, PHT and hematochezia without hematemesis.^3^ Yet, several authors have reported cases similar to ours, where patients had not been operated on.^11^ Small bowel varices‐related bleeding was reported to be massive in several cases with patients presenting with hemodynamic instability and hemoglobin levels of 6 g/dL or lower.^2^, ^5^, ^8^, ^11^, ^12^ The bleeding‐related mortality was estimated in a recent study to range from 5.3% to 18.4%.^10^

Exploring the small bowel can be performed with contrast imaging, nuclear studies, angiography or with endoscopic procedures such as capsule endoscopy or push enteroscopy. In this context, CT scan or angiography are thought to be the most suitable as they can be immediately performed with no required preparation which make them appropriate for emergency cases. On the other hand, both methods rarely show contrast extravagation which was the case for our patient.^8^ In fact, in most published cases, CT scan does identify the jejunal varices but fail to document the bleeding.^2^, ^11^, ^12^ Although capsule endoscopy (CE) and deep enteroscopy are currently considered procedures of choice to explore obscure GI bleeding, their use is limited by the necessity of a skilled endoscopist and the fact that no therapeutic interventions can be performed with CE. In addition, jejunal varices can be mistaken during these endoscopic procedures for angiodysplasia lesions. Hypotheses for this confusion include the presence of intravascular hemangiomatous components or intravascular thrombosis.^13^

When it comes to clinical management, the first step usually consists in resuscitation measures and correction of anemia. Although their role has not yet been established in small intestine bleeding, somatostatin analogs have been used as a temporizing measure by several authors as well as ourselves.^10^, ^14^ To date, no evidence‐based guidelines dealing with jejunal varices treatment have been published. Difficult to localize and reach with limited published data, management of small bowel varices remains challenging. The choice of therapeutic procedure mainly depends on the patient hemodynamic state, the clinician's experience and the available technical platform. For our patient, local treatment was not possible seen that enteroscopy was not available in our institution. Thus, we opted for coil embolization. The procedure is a safe option to treat these patients, it consists in catherizing and embolizing the vein leading to jejunal varices. However, this procedure can be difficult when the varices net is extended.^15^ The outcomes of coil embolization in this case were satisfying on both the short and long term. These results are in accordance with an American study analyzing the outcomes of percutaneous treatment in patients with ectopic GI varices bleeding.^15^ It is worth noting, however, that the study reported a recurrence rate of 11% during the 3 days following coil embolization. Although embolization is usually efficient to stop the bleeding in the short term, the observed high recurrence rates are expected as embolization does not assure any decompression of the portal venous system. Embolization complications are rare with a reported incidence of 3%.^16^ Major complications include hemoperitoneum, Enterobacter sepsis, coil migration, acute renal and liver failure, and intrasplenic hematoma for patients undergoing percutaneous transsplenic catherization.^16^, ^17^

Balloon‐occluded retrograde transvenous obliteration (BRTO) is another radiological intervention that can be used in ectopic varices‐related bleeding.^1^ BRTO allows the occlusion of the afferent and efferent vessels in addition to the varices causing the bleeding. Yet, this technique can lead to a significant increase in the portosystemic pressure gradient with development or progression of esophageal varices.^18^

Decompressive treatments include TIPS and surgical portosystemic shunting seem to be effective in this context. In fact, TIPS is recommended by the American Association for the Study of the Liver as the most suitable preventive treatment for patients with recurrent hemorrhage.^19^ On the contrary, surgical decompressive interventions indications are currently considered a salvage treatment in cases of non‐availability or failure of endoscopic and radiological treatments.^20^ The procedure consists in creating portosystemic, mainly distal splenorenal shunt, or porto‐portal shunts and performing regional devascularization.^20^

However, both approaches can only be offered to patients with a relatively preserved liver function and can only be performed by experienced surgeons or radiologists. In addition, surgery and TIPS are associated with several complications and a high mortality exceeding in some cases the risk of fatal hemorrhage.^15^ In non‐cirrhotic patients, decompressive techniques seem to be the optimum choice. In 2019, an American team performed transhepatic portal vein stenting as a decompressive treatment in a non‐cirrhotic patient with a high‐grade portal stenosis. This led to immediate hemorrhage control and no recurrence was documented during a 3‐month follow‐up period.^12^

Endoscopic approach in this context might be appropriate in case of small, non‐extended varices.^1^ Both band ligation and sclerotherapy were reported to be efficient to achieve immediate hemostasis with better results observed with the latter. Both techniques are technically challenging requiring experienced endoscopists and are associated with high recurrence rates.^21^ Yet, the endoscopic approach remains very valuable during the follow‐up to ensure variceal eradication and for the surveillance even when the initial bleeding episode is controlled with radiological interventions.^1^ The main side effects of endoscopic local treatments are perforation and varices ulcers‐related bleeding.^14^

To sum up, jejunal variceal bleeding is a rare but life‐threatening entity. It should be considered in patients with PHT and obscure bleeding even in absence of surgical history. The scarcity of data related to the different treatment options makes it hard to make any conclusions or recommendations concerning their efficacy. When compared to the endoscopic approach, coil embolization seems to be a more available and less difficult treatment. Yet, more studies remain warranted to confirm its short‐ and long‐term outcomes.

## AUTHOR CONTRIBUTIONS

Sahar Nasr and Wafa Dahmani involved in manuscript drafting. Hanene Jaziri and Wafa Ben Ameur involved in critical review of the manuscript. Yesser Becheikh involved in data collection and treatment of the patient. Nour Elleuch and Ali Jmaa involved in final approval of the manuscript.

## FUNDING INFORMATION

No grants were obtained from any institutions for this research paper.

## CONFLICT OF INTEREST

The authors declare that they have no competing interests.

## ETHICAL APPROVAL

Ethical committee's approval is not needed for case reports in Tunisia.

## CONSENT

Written informed consent was obtained to publish this case.

## Data Availability

The data available were included in the manuscript.
